# Microalgae for freshwater arsenic bioremediation: examining cellular toxicity, bioconcentration factor and eluding an alternative arsenic detoxification pathway

**DOI:** 10.1007/s13205-024-03977-w

**Published:** 2024-04-10

**Authors:** Wenn Wenn Tang, Su Chern Foo

**Affiliations:** 1 School of Science, Monash University Malaysia, Jalan Lagoon Selatan, 47500 Bandar Sunway, Selangor Darul Ehsan Malaysia; 2 Monash University Malaysia, Tropical Medicine and Biology Multidisciplinary Platform, Jalan Lagoon Selatan, 47500 Bandar Sunway, Selangor Darul Ehsan Malaysia

**Keywords:** Cyanobacteria, *Chlorella*, Phosphate, Toxicity, Metabolism

## Abstract

Microalgae are photoautotrophic organisms in freshwater systems known to uptake and bioremediate arsenic, a heavy metal. In this study, we compared the growth and arsenic uptake of two microalgae strains, *Nostoc* and *Chlorella*, to determine their suitability for arsenic bioremediation. As compared to the control, our results showed that treatment with As (III) enhanced the *Nostoc* growth by approximately 15% when grown in the absence of phosphate. The highest bioconcentration factor of *Nostoc* at this treatment was 1463.6, whereas 0.10 mg L^−1^ As (V) treatment improved the *Chlorella* growth by 25%, in the presence of phosphate. However, arsenic uptake reduced from 175.7 to 32.3 throughout the cultivation period for *Chlorella***.** This suggests that *Nostoc* has an upper advantage in the bioremediation of arsenic as compared to the *Chlorella* strain. To gain insights into the potential of *Nostoc* in arsenic bioremediation, we further conducted SEM analysis on the vegetative cell surface. The SEM results showed that As (III) disrupted the *Nostoc* vegetative cell surface and structure. Further to this, pathway analysis and polymerase chain reaction (PCR) were conducted to identify the potential arsenic pathway regulated by *Nostoc*. The primary As (III)-related pathways elucidated include the arsA transporter and arsD complex that require ATP and As (III) methylation to *S*-adenosylmethionine. The phosphate deficiency condition resulting in the inability to generate ATP caused As (III) could not be excreted from the *Nostoc* cells, potentially contributing to the high arsenic concentration accumulated under phosphate-depleted conditions. These insights contribute to understanding the efficacy of microalgae strains in freshwater arsenic bioremediation.

## Introduction

Arsenic (As) is a non-essential heavy metal ranked first on the list of poisons by the United States Environmental Protection Agency (EPA). It is a natural element widely distributed in the Earth’s crust (Reid et al. 2020). Various studies have provided evidence that industrial activities including mining, smelting, and burning of fossil fuels, led to the contamination of soil and water due to the release of arsenic (Wang et al. 2015). Arsenic concentrations exposed to humans are considered low at 0.6 mg L^−1^ (National Research Council (U.S.) (1999)). However, it is crucial to recognize that even low levels of arsenic exposure can result in several health complications for individuals, affecting the kidney, liver, and neurological systems (Ong et al. 2013; Shanab et al. 2012).

Arsenic manifests in various forms referred to as species, encompassing organic variants such as monomethylarsonic acid (MMA) and dimethyl arsenic acid (DMA) (Schreiber and Cozzarelli 2021). The inorganic form of arsenic exists predominantly in two oxidation states: trivalent arsenic, i.e., arsenite As (III), and pentavalent arsenic, i.e., arsenate As (V) (Yan et al. 2019). Several studies confirmed that microalgae possess the capability to uptake high levels of arsenic. Microalgae uptake As (III) into cells through aquaglyceroporins (Zhang et al. 2014) and uptake As (V) through the phosphate transport channel (Ferrari et al. 2013). Intriguingly, while arsenic is highly toxic to humans, microalgae exhibit a remarkable resilience to elevated arsenic concentration, enabling their survival under such conditions.

Phosphate is an important macronutrient essential for microalgae growth, playing a role in forming phospholipids, DNA, RNA, and ATP for metabolic pathways and energy transfer (Dyhrman 2016; Yaakob et al. 2021). Due to the structural similarity of phosphate and As (V), the uptake of As (V) into cells will be suppressed by phosphate (Zhang et al. 2014), failing As (V) to incorporate into cells (Ferrari et al. 2013). Moreover, conversion of As (III) to As (V) through oxidation occurs more rapidly under high phosphate levels (Miyashita et al. 2015), leading to an increase of As (V) over time in phosphate-rich conditions compared to phosphate-limited conditions (Zhang et al. 2014). As per our current knowledge, there is no report on the phosphate affecting the uptake or metabolism of As (III) in microalgae cells.

*Nostoc*, the cyanobacteria and *Chlorella*, the green microalgae, have demonstrated the ability to uptake high arsenic concentrations (Patel et al. 2021; Higashi et al. 1985). In this study, an assessment of the growth and arsenic uptake by two microalgae strains *Nostoc* MUM003 and *Chlorella* MUM002 strain was conducted, with a particular focus on phosphate as an influence factor to compare the suitability of the microalgae strains in arsenic bioremediation. The findings revealed that under phosphate-depleted conditions, *Nostoc* performed better compared to the *Chlorella* strain both in terms of growth and arsenic uptake. A detailed examination of the vegetative cells of *Nostoc* was performed to identify the effect of As (III) on the cell surface. Through the gene pathway analysis, 3 main pathways involving As (III) in *Nostoc* cells were predicted, and the possible reason for the phosphate-depleted condition leading to a higher arsenic concentration accumulated in *Nostoc* cells was hypothesized. The overall results indicate that *Nostoc* can survive and uptake arsenic, although damage on the cell surface was observed, and a new hypothesis of arsenic accumulation influenced by phosphate was hypothesized.

## Materials and methods

### Strains and culture conditions

*Nostoc* NIES-2111_MUM004 strain (SRR27732368) and *Chlorella sorokiniana*_MUM002 strain (SRR27765439) were collected from the freshwater lakes around Peninsular Malaysia. Collected samples were washed and cultivated on fresh BG-11 agar to obtain a single colony (Parvin et al. 2007). Pure microalgae cultures were grown and maintained in BG-11 medium with continuous monitoring to ensure there was no contamination. To prevent sedimentation, cultures were shaken twice daily.

### Arsenic and phosphate treatment

Both microalgae strains were cultivated in fresh BG-11 medium for 14 days to reach the exponential phase. The cells were harvested via centrifugation and washed with autoclaved Milli-Q water. Washed cells were pre-treated in a phosphate-depleted BG-11 medium for 5 days to eliminate phosphate present in the microalgae cells. The initial cell density for the subsequent experiment was approximately 5 × 10^5^ cells mL^−1^ for *Nostoc* and 1 × 10^4^ cells mL^−1^ for *Chlorella*, reflecting the varying growth rates of both strains. Both microalgae cells were subjected to the same conditions stated in Table 1. The cultivation took place at 25 °C, with a light–dark cycle of 16 h of light and 8 h of darkness, maintaining the illumination at 2500 lx (Minhas et al. 2020). To prevent sedimentation, all cultures were shaken twice per day. *Nostoc* was cultivated for a 30-day period, while *Chlorella* was incubated for 7 days.

**Table 1:** Arsenic and phosphate concentration

Arsenic concentration (mg L^−1^)		Phosphate concentration (mg L^−1^)
As (III)	As (V)	
0.01	0.00	0.00
0.10	0.00	
1.00	0.00	
0.00	0.01	
0.00	0.10	
0.00	1.00	
0.01	1.00	
0.10	0.10	
1.00	0.01	
0.01	0.00	0.24
0.10	0.00	
1.00	0.00	
0.00	0.01	
0.00	0.10	
0.00	1.00	
0.01	1.00	
0.10	0.10	
1.00	0.01	

### Growth measurement

The typical growth rate of *Nostoc* is 30 days (Spencer et al. 2011) and Chlorella is 7 days (Gitau et al. 2021). Therefore, the sampling frequency for both microalgae strains was decided at 3-day intervals for *Nostoc* and 24-h intervals for *Chlorella*. Approximately, 15 mL of microalgae cultures were collected, and the growth of the microalgae strains was measured through the biomass dry weight method.

A 1.2 µm nylon filter (Bioflow, Malaysia) was used for the biomass dry weight measurement of both microalgae cultures. The nylon filter was pre-washed with distilled water to eliminate any dust and air bubbles adhering to the filter paper. Subsequently, the filter membrane was dried at 60 °C for 24 h. The filter membranes were transferred into a desiccator to cool down and the weight of the filter papers was recorded at least 3 times until a constant mass was obtained. For the measurement of biomass dry weight, 10 mL of culture was collected and filtered through the pre-washed filter membrane. The filter membrane was subjected to drying, and re-weight unto a constant mass was obtained (Ratha et al. 2016).

### Intracellular arsenic analysis

Approximately, 50 mL of the microalgae cultures was collected and filtered using a 1.2 um membrane filter (Bioflow, Malaysia). The filtrates were retained for extracellular arsenic concentration measurement. The cells on the membrane filter were rinsed with 20 mL of arsenic-free BG-11 medium and digested using 8 mL of 25% HNO_3_ for 30 min at room temperature. The digest was heated in a microwave at 90 W for 5 min and diluted to a 10% HNO_3_ concentration using Milli-Q water (Levy et al. 2005). Both the filtrates and digested cells were analyzed using the ICP-OES instrument to determine the total arsenic concentration. The total intracellular arsenic concentration was calculated as *Ci*, where C1 represented the concentration detected by the ICP-OES instrument:

$$Ci\frac{C1}{12}\times 50$$

### Data treatment and modeling

Statistical analysis was performed using GraphPad Prism 9.5.1 to analyse the microalgae growth and arsenic uptake concentrations data. The Normality and Lognormality tests were performed with the Shapiro–Wilk test to identify the normal distribution of the results and obtain the P-value. Two-way ANOVA analysis was performed to identify the significant differences between the treatments.

The specific growth rates were calculated as *µ*_s_, where N_1_ and N_2_ demoted the cell density at time t_1_ and t_2,_ respectively:

$$\upmu s\hspace{0.17em}=\hspace{0.17em}Ln({N}_{2})-Ln({N}_{1})/({t}_{2}-{t}_{1})$$

To compare the effect of arsenic on cell growth to the control, the relative growth rate percentage (%) was calculated by normalizing *µ*_s_ to the control (*µ*_0_). The improvement or reduction percentage (%) was used as the biological response to estimate the arsenic toxicity:

$$\mathrm{\% }=\upmu s/\upmu 0$$

Due to the difference in arsenic treatment concentration, the bioconcentration factor (BCF) was calculated to identify the direct uptake or absorption of arsenic from the medium by microalgae cells, where C_i_ showed the intracellular arsenic concentration measured, and C_0_ represented the initial arsenic concentration fed:

$$BCFs=\frac{(Ci)}{C0}$$

Extracellular arsenic percentage was calculated As (%), where C_0_ and C_e_ showed the initial concentration fed and extracellular arsenic concentration measured, respectively:

$$\mathrm{\%}=\frac{(C0-Ce)}{C0}{\text{X}}100$$

### *Nostoc* surface structural analysis

1 mL *Nostoc* culture aliquot was collected on the 24th day of cultivation with 1.00 mg L^−1^ As (III) treatment without phosphate, which exhibited the highest arsenic uptake. A drop of microalgae culture was placed onto a cover slip and chemically fixed using 4% glutaraldehyde for 12 h. Following fixation, the samples were rinsed with PBS buffer and repeated twice. The microalgae samples were subsequently dehydrated in a graded series of ethanol (30, 50, 75, 85, 95, and 100%) for 3-min intervals each, and dried in a desiccator overnight (Huang et al. 2013). Gold was selected as the coating material to coat the microalgae sample. The samples were loaded onto the SEM instrument to examine the surface structural alterations and SEM–EDX was employed to detect the presence of arsenic on the microalgae’s surface.

### DNA extraction and whole-genome sequencing

DNA of *Nostoc* was extracted using DNeasy PowerSoil Pro Kits (Qiagen, Germany) according to the manufacturer’s protocol. The DNA was sequenced through the Illumina MiSeq sequencer in the Genomics Facility, Monash University Malaysia. The whole-genome sequence (WGS) was assembled and annotated through the Galaxy tool (https://usegalaxy.org/).

### Pathway analysis

The proteins, RNA, genes, and compounds presented in the *Nostoc* genome were predicted using the standalone Biocyc Pathway tool v27.0 (https://biocyc.org/). To identify the overview pathways presented in *Nostoc*, the database of the *Nostoc* was first built using the PathoLogic function with the annotated gene bank and fna file downloaded from the Galaxy tool result. The arsenic-related genes and pathways were viewed from the overview figure or the compound search bar.

### Primer design

Arsenic-related genes were identified and primers were designed to identify the regulation of the targeted genes. The sequence from the FastA file in the nucleic acid sequence was selected and the Primer3Plus tool (https://www.bioinformatics.nl/cgi-bin/primer3plus/primer3plus.cgi) was used to design all targeted primers. Designed primers were purchased from Apical Scientific (Malaysia).

#### RNA extraction and PCR analysis

The RNA of *Nostoc* cells treated with 1.00 mg L^−1^ without phosphate on day 24 and control were extracted using the RNA extraction kit (Zymo Research, United States). The concentration and purity of the RNA were checked using a NanoDrop spectrophotometer (Thermo Fisher Scientific, Malaysia) and 1% agarose gel (Vivantis Technologies, Malaysia). Pure RNA was further analysed through PCR using REDiant II PCR Master Mix (Apical Scientific, Malaysia), to identify the presence of targeted genes.

## Results

### Biomass dry weight of microalgae treated with arsenic

#### Arsenic increases *Nostoc* biomass when lack of phosphate

**Fig. 1:**
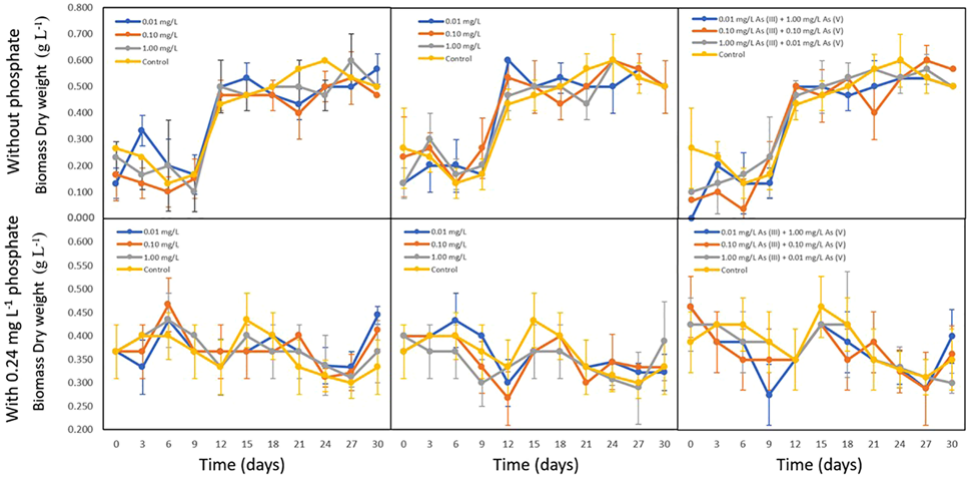
Biomass dry weight of *Nostoc* treated in phosphate-depleted BG-11 medium with 0.01 mg L^−1^, 0.10 mg L^−1^, and 1.00 mg L^−1^ of As (III), As (V), and mixed arsenic species, compared to the same treatments supplied with 0.24 mg L^−1^ phosphate (P < 0.05). Concentrations (mg L^−1^) are noted by the symbols of the legend superimposed on each graph. *Nostoc* showed average higher biomass dry weight for all arsenic treatments when there is a lack of phosphate during the exponential phase (Day 12), whereas under phosphate-supplied conditions, only As (III) and 0.01 mg L^−1^ As (V) showed higher biomass during the initial cultivation phase

**Fig. 2:**
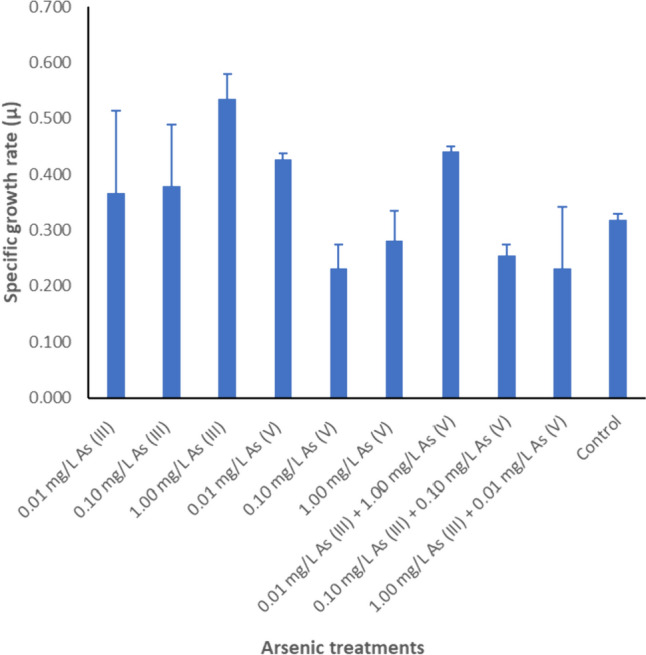
Specific growth rate (µ) of *Nostoc* treated in phosphate-depleted medium with 0.10 mg L^−1^, 0.10 mg L^−1^, and 1.00 mg L^−1^ concentrations of As (III), As (V), and mixed arsenic species in the absence of phosphate during the exponential phase (P < 0.05). It is clear that *Nostoc* showed a higher specific growth rate under 1.00 mg L^−1^ As (III) treatment. The 0.10 mg L^−1^ As (V) showed the lowest specific growth rate

The results in Fig. 1 revealed that arsenic has the ability to improve the biomass of *Nostoc.* Notably, *Nostoc* cultivated without phosphate showed a higher biomass dry weight compared to the control when treated with 9 different arsenic treatments (P < 0.05). The control treatment without phosphate exhibits a higher biomass in the late stationary phase. The late exponential phase on day 12 demonstrated that single As (III) species with 0.01 mg L^−1^ and 1.00 mg L^−1^ concentration increased the biomass of *Nostoc*. In contrast, lower concentrations of single As (V) species induce higher biomass of *Nostoc* compared to higher As (V) concentrations. In mixed arsenic treatments with the lack of phosphate, the same arsenic species ratios and treatments with a higher As (V) ratio resulted in an improvement of the biomass compared to treatments with a higher As (III) ratio. Comparing all arsenic treatments to the control (Fig. 2), it was observed that 1.00 mg L^−1^ As (III) exhibited the highest specific growth rate during the exponential phase when there was a lack of phosphate. This was followed by mixed arsenic treatments with a higher As (V) ratio and 0.01 mg L^−1^ As (V) treatments.

When subjected to treatments containing 0.24 mg L^−1^ phosphate, all As (III) concentrations contributed to a higher biomass during initial cultivation days and late exponential phase to decline phase. Among all As (III) treatments, the 0.10 mg L^−1^ concentration induces the highest biomass, followed by 0.01 mg L^−1^ and 1.00 mg L^−1^ concentration. In contrast, As (V) treatments showed only the lowest concentration improved the biomass of *Nostoc* from day 3 to day 12, while all treatments showed a reduction in biomass compared to the control from the exponential phase to the decline phase. A similar trend was observed for mixed arsenic species treatment, where the presence of mixed arsenic led to either a similar or a reduction of *Nostoc* biomass throughout the 30-day cultivation period.

#### Arsenic reduces *Chlorella* biomass when lack of phosphate

**Fig. 3:**
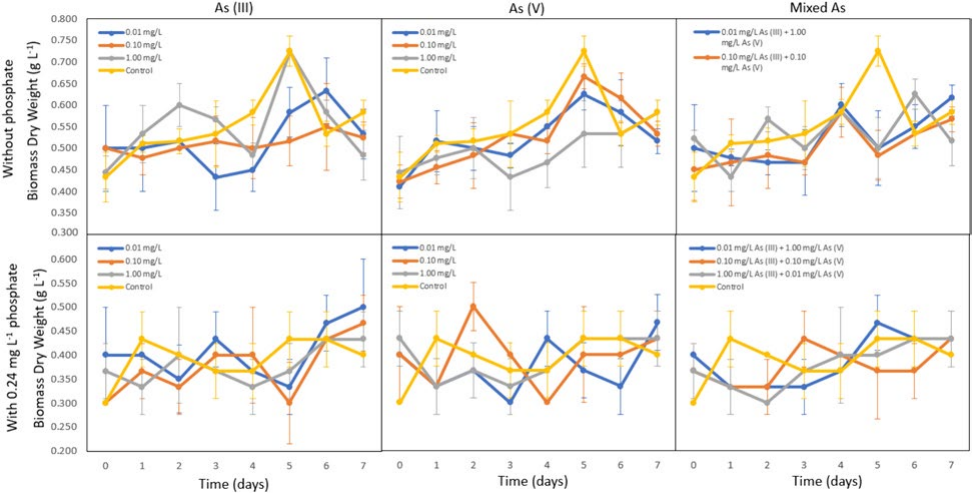
Biomass dry weight of *Chlorella* treated in phosphate-depleted BG-11 medium with 0.01 mg L^−1^, 0.10 mg L^−1^, and 1.00 mg L^−1^ of As (III), As (V), and mixed arsenic species, compared to the same treatments supplied with 0.24 mg L^−1^ phosphate (P < 0.05). Concentrations (mg L^−1^) are noted by the symbols of the legend superimposed on each graph. *Chlorella* showed average lower biomass dry weight for all arsenic treatments when there is a lack of phosphate during the exponential phase (Day 12) except 1.00 mg L^−1^ As (III) during the initial cultivation period, whereas, under phosphate-supplied conditions, 0.10 mg L^−1^ As (V) showed a higher biomass compared to the control

The results in Fig. 3 revealed that *Chlorella* increased in biomass from day 1 to day 5 when phosphate was absent. However, across all 9 treatments, *Chlorella* averagely reduced the biomass compared to the control. Specifically, the 0.01 mg L^−1^ and 0.10 mg L^−1^ concentrations for both As (III) and As (V) exhibited similar growth patterns. On the other hand, 1.00 mg L^−1^ As (III) increases the biomass during the initial cultivation phase. In contrast, 1.00 mg L^−1^ As (V) showed a reduction in the *Chlorella* biomass throughout the 7-day cultivation period. For mixed arsenic species treatment, arsenic species with the same ratio and treatment with higher As (V) concentration showed a similar pattern. This indicates that As (V) has a higher influence on the growth compared to As (III) when there is a presence of different arsenic species. The low As (III) concentration does not significantly increase or reduce the biomass of *Chlorella*. Conversely, treatment with higher As (III) concentration showed higher biomass during the initial and the end of the cultivation period compared to the control and other mixed arsenic treatments. This indicated that As (III) can improve the growth of *Chlorella* but only during the initial cultivation days.

Treatment with 0.24 mg L^−1^ phosphate supplied showed that arsenic generally reduces the biomass of *Chlorella*. Notably, 0.01 mg L^−1^ and 0.10 mg L^−1^ As (III) increase the biomass during mid-cultivation phase. At the concentration of 0.10 mg L^−1^, As (V) enhances the biomass of *Chlorella,* but lower and higher As (V) concentrations reduce the growth. For mixed arsenic concentration, arsenic species with the same ratio increase the biomass during the mid-cultivation period, whereas treatment with higher As (V) concentration increases the biomass during the late treatment phase.

#### 1.00 mg L^−1^ As (III) concentration increases microalgae biomass when lack of phosphate

**Fig. 4:**
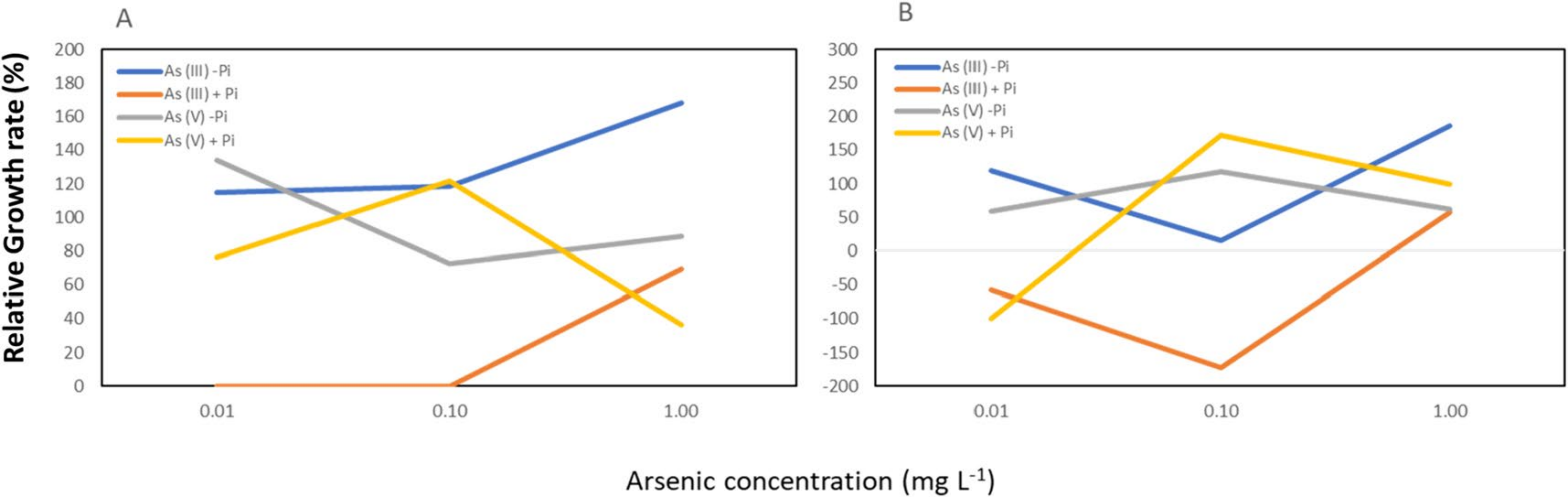
Relative growth rate percentage (%) of *Nostoc* (A) and *Chlorella* (B) treated with 0.01 mg L^−1^, 0.10 mg L^−1^, and 1.00 mg L^−1^ As (III) and As (V), with the absence or presence of phosphate during the exponential phase. *Nostoc* showed the highest relative growth rate percentage under 1.00 mg L^−1^ As (III) without phosphate, compared to 0.01 mg L^−1^ and 1.00 mg L^−1^ arsenic with or without phosphate, whereas *Chlorella* showed a lower relative growth rate percentage for 0.10 mg L^−1^ As (III) when there is the presence of phosphate

The findings highlighted that *Nostoc* achieved the highest biomass during the exponential phase on day 12, while *Chlorella* exhibited the highest biomass on day 5. Relative growth rate comparing microalgae strains treated with different arsenic concentrations during the exponential phase to the control showed that there is no significant difference in biomass of *Nostoc* treated with 0.01 and 0.10 mg L^−1^ As (III) with the presence of phosphate (Fig. 4). However, the growth rate showed a significant increase by 68% when treated with 1.00 mg L^−1^ As (III) compared to the growth rate of control treatment. Notably, the growth rate of *Nostoc* is higher when there is a lack of phosphate compared to phosphate-supplied conditions. Furthermore, it was observed that the treatment with 0.10 mg L^−1^ As (V) significantly reduced the biomass of *Nostoc* by 63% compared to 0.01 mg L^−1^ without the presence of phosphate, but it led to an increase in the biomass when phosphate was supplied.

The relative growth rate percentage of *Chlorella* treated with As (III) showed a similar trend where 0.10 mg L^−1^ As (III) declined the growth rate, whereas 0.10 mg L^−1^ As (V) increased the biomass. Figure 3 proves that 1.00 mg L^−1^ As (III) could enhance the biomass of *Chlorella* compared to 0.10 mg L^−1^ As (III) treatment.

### Arsenic uptake by microalgae

**Fig. 5:**
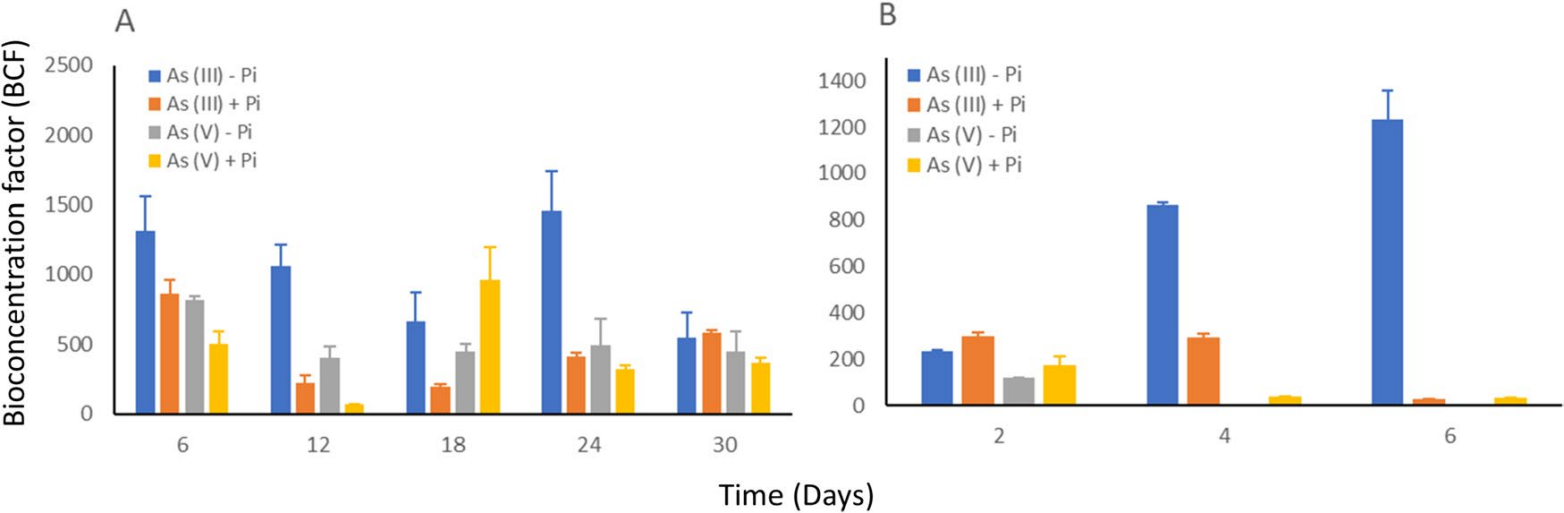
Bioconcentration factor of *Nostoc* (A) and *Chlorella* (B) treated with 1.00 mg L^−1^ As (III) or As (V) with or without 0.24 mg L^−1^ phosphate (P < 0.05). BCF values greater than 1000 are considered to be hyperaccumulators. *Nostoc* treated with As (III) without phosphate showed averagely higher BCF values, where the highest value exceeded 1500 on day 24. *Chlorella* showed the highest BCF values under As (III) treatment without phosphate, and both As (V) treatments with and without phosphate showed significantly low BCF values

*Nostoc* showed the ability to uptake both As (III) and As (V) through bioconcentration factor under phosphate-depleted and supplied conditions (Fig. 5) (P < 0.05). Notably, *Nostoc* uptake high As (III) concentrations with a BCF value of 1463.6 when phosphate is absent over the entire 30-day cultivation period. However, arsenic uptake treated with As (III) with phosphate showed higher BCF values during initial cultivation days, compared to the stationary phase on day 18. In the case of the As (V) treatment, *Nostoc* showed a lower BCF value of 66 compared to As (III), with the exception of the As (V) treatment with phosphate on day 12. Moreover, *Nostoc* uptake higher arsenic percentage treated with As (V) without phosphate compared to conditions with phosphate. The overall results showed that the highest arsenic uptake by *Nostoc* is As (III) without phosphate on day 24, while the lowest arsenic uptake is As (V) with the presence of phosphate on day 12.

*Chlorella* demonstrated the ability to uptake arsenic when treated with As (III) with a BCF value of 1231.6, while the least arsenic uptake was observed when treated with As (V) where almost no arsenic was detected (Fig. 5) (P < 0.05). The arsenic uptake by *Chlorella* increased gradually under conditions of As (III) treatment without phosphate, while As (III) treatment with phosphate and As (V) treatments showed a reduction in arsenic uptake over the cultivation period. Chlorella can uptake higher As (III) after 6 days of cultivation, however, the uptake of As (V) under the presence of phosphate remained low.

Comparing the arsenic uptake of both microalgae strains through the BCF values, *Nostoc* possessed a significant capability in arsenic accumulation compared to *Chlorella*. The overall BCF values of *Nostoc* in the uptake of both As (III) and As (V) were significantly higher compared to the BCF values of *Chlorella*. The similarity between both strains was the uptake or BCF values were higher for As (V) without phosphate treatment during initial cultivation phases, whereas higher BCF values for As (III) without the presence of phosphate after several days of cultivation. These results concluded that *Nostoc* can uptake a higher arsenic concentration compared to *Chlorella* supplied with the same treatment condition.

### Arsenic removal percentage

The arsenic removal percentage for both *Nostoc* (Day 30) and *Chlorella* (Day 7) is shown in Table 2. Arsenic treatment with 0.01 mg L^−1^ concentration revealed that *Nostoc* achieved the highest arsenic removal percentage (99.40%) when phosphate was absent, followed by *Chlorella* which removed 80.37% arsenic under the same treatment conditions. Conversely, *Chlorella* showed the highest arsenic removal percentage (92.90%) when subjected to 0.01 mg L^−1^ arsenic treatment with phosphate, while *Nostoc* showed the highest arsenic removal percentage (75.24%) treated with 1.00 mg L^−1^ arsenic without phosphate. In consideration of the initial arsenic concentration treated to the microalgae strains, *Nostoc* showed the highest arsenic concentration removed from the medium under 1.00 mg L^−1^ As (III) without phosphate.

**Table 2:** Arsenic removal percentage by *Nostoc* and *Chlorella* on Day 30 and Day 7

Arsenic species	Arsenic concentration (mg L^−1^)	Arsenic removal percentage (%)			
		*Nostoc*		*Chlorella*	
		Without phosphate	With 0.24 mg L^−1^ phosphate	Without phosphate	With 0.24 mg L^−1^ phosphate
As (III)	0.01	61.22 ± 15.36	69.38 ± 0.88	72.05 ± 16.66	59.23 ± 13.51
	0.10	59.76 ± 6.93	89.68 ± 11.76	44.44 ± 2.81	92.90 ± 1.76
	1.00	75.24 ± 28.66	67.68 ± 3.52	21.76 ± 5.54	39.83 ± 5.70
As (V)	0.01	99.40 ± 0.00	65.00 ± 21.21	80.37 ± 14.60	78.95 ± 2.28
	0.10	39.72 ± 7.34	90.00 ± 1.41	42.98 ± 11.25	73.95 ± 11.79
	1.00	54.72 ± 43.39	74.91 ± 4.59	17.74 ± 0.46	53.69 ± 2.33

### Ultrastructural analysis by scanning electron microscopy (SEM)

**Fig. 6:**
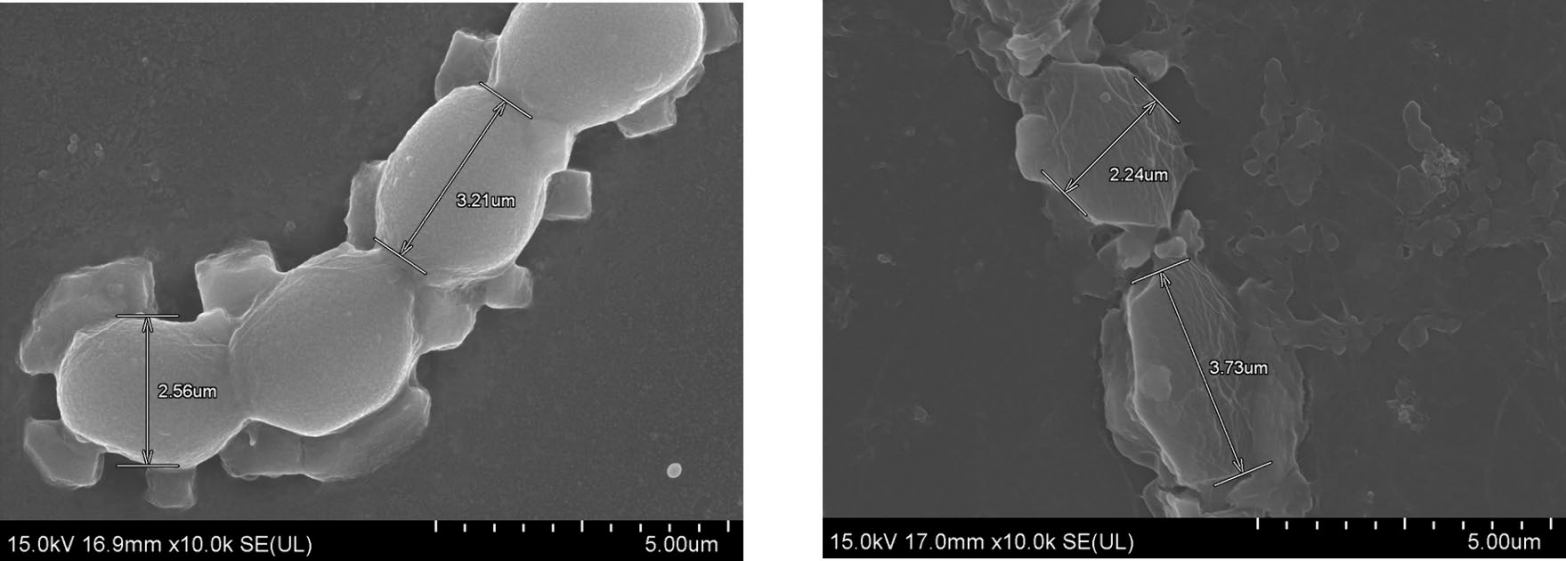
Overall figure of *Nostoc* under control treatment (left) and 1.00 mg L^−1^ As (III) treatment (right) viewed under SEM. It can be seen that the cell surfaces of the As-treated cells exhibited greater signs of rupture, roughness with rigid textures, and corrugation when compared to the smooth surfaces of the control cells. Although the cell surface differs for both with and without treatment cells, the cell size of *Nostoc* does not exhibit substantial differences

**Fig. 7:**
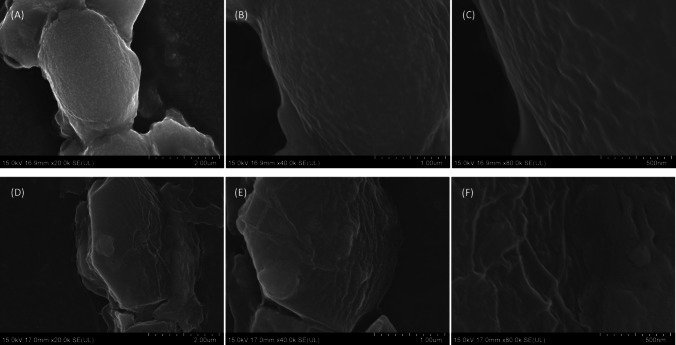
Surface structure of *Nostoc* vegetative cell under control treatment with 20 k × magnification (**A**), 40 k × magnification (**B**), 80 k × magnification (**C**) and 1.00 mg L^−1^ As (III) treatment with 20 k × magnification (**D**), 40 k × magnification (**E**), 80 k × magnification (**F**) viewed under SEM. *Nostoc* treated with 1.00 mg L^−1^ As (III) (**D**, **E**, **F**) showed changes in cell surface structure, with a more ruptured cell surface compared to the control. This indicates that As (III) treatment causes damage to the *Nostoc* vegetative cell surface

**Fig. 8:**
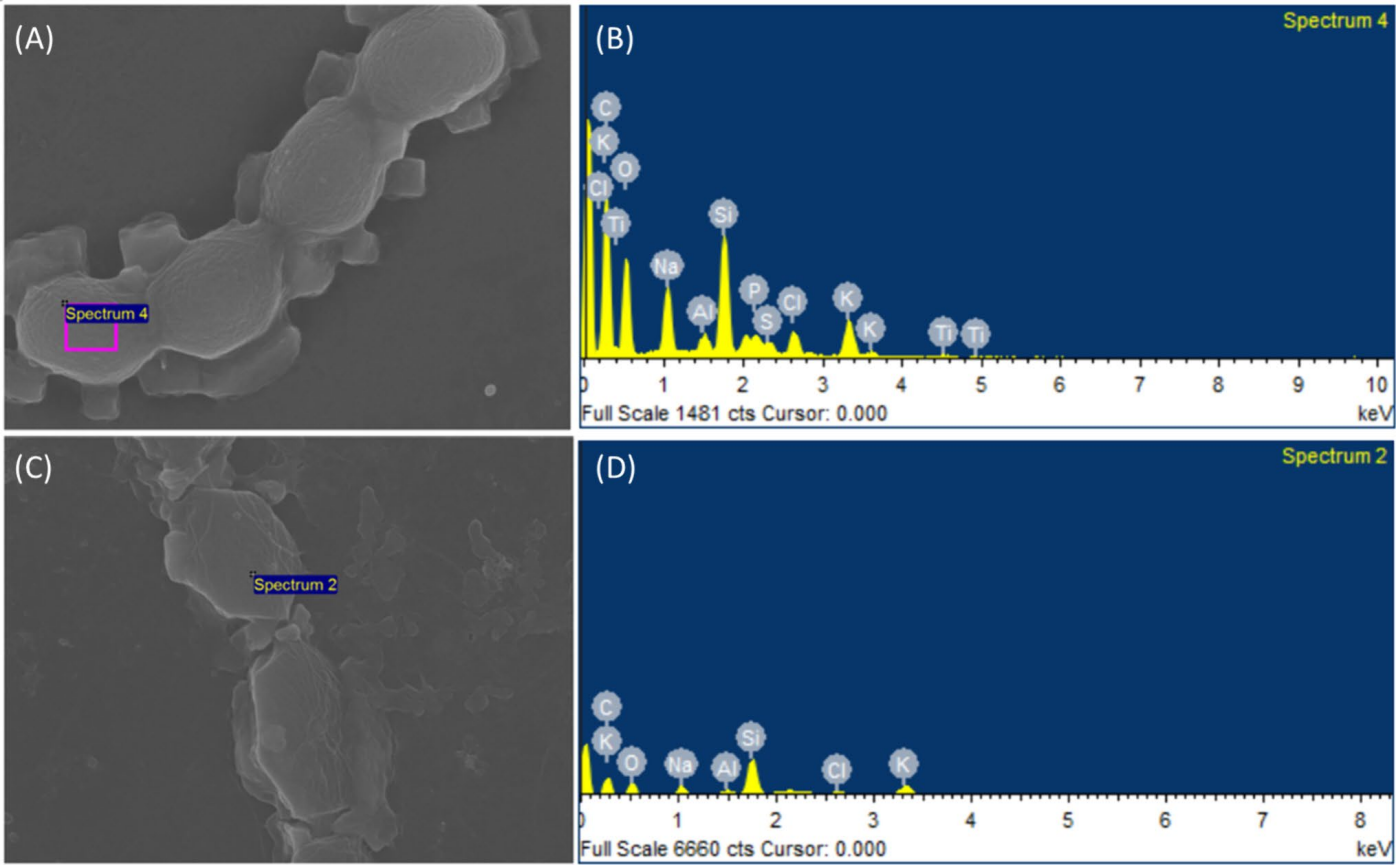
SEM–EDX image and results for *Nostoc* under control (**C**, **D**) treatment, and cells treated with 1.00 mg L^−1^ As (III) (**C**, **D**). Both control and treated cells do not show the presence of arsenic in the SEM–EDX result indicating As (III) does not adsorb on the surface of *Nostoc* cells although damage was observed

As *Nostoc* showed the ability to enhance the growth rate, and uptake and remove the highest arsenic concentration under 1.00 mg L^−1^ As (III) without phosphate, the vegetative cell surface was examined to identify the arsenic concentration adsorb on the cell surface and the effect of arsenic on the structural changes. The ultrastructural surface of *Nostoc* viewed under SEM (Fig. 6) indicates that there are no significant differences in terms of vegetative cell size for both the control and As (III)-treated cells. However, although there are no differences in terms of vegetative cell size, the vegetative cells with arsenic treatment exhibited rougher, extensively damaged, and disrupted cell surfaces (Fig. 7) in comparison to the control cells. The treated cells displayed rougher surfaces with more wrinkles compared to the control cells with smooth surfaces. In addition, the cell shape with arsenic treatment showed damages compared to the round, spherical shape of the control cells. The results in Fig. 8 indicated that there is no detectable arsenic on the surface of *Nostoc* for both control and treated cells. Furthermore, aside from arsenic, most of the elements detected on the control cell surface showed a lower concentration in arsenic-treated cells, including carbon, potassium, and sodium.

### As (III)-related pathways in *Nostoc*

**Fig. 9:**
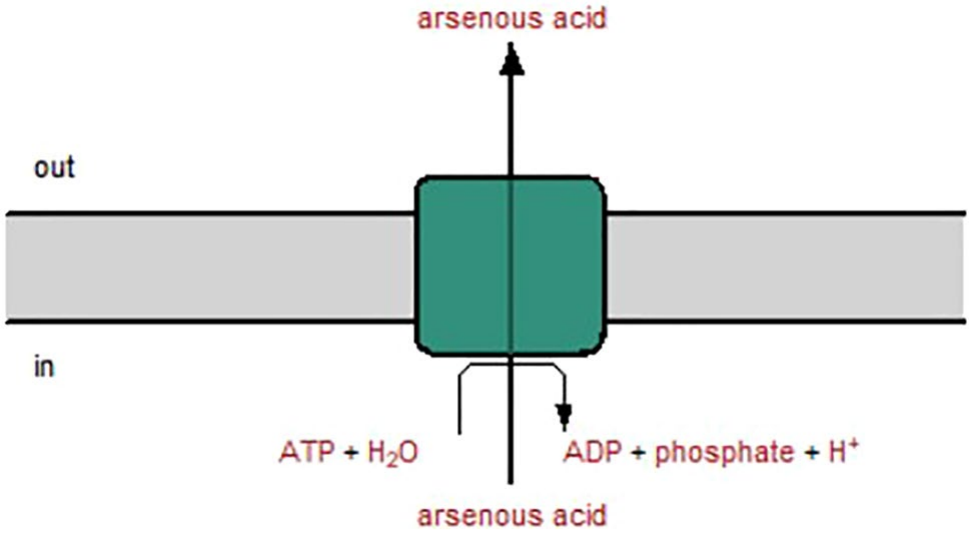
Arsenical pump-driving ATPase (arsA) transporter pathway generated through the Pathway tool. The arsA pathway excretes As (III) out from the *Nostoc* cell by hydrolyzing ATP to ADP. Under the phosphate-depleted condition, ATP were not readily available thus this pathway were either inhibited or downregulated to activate the excretion of As (III)

**Fig. 10:**
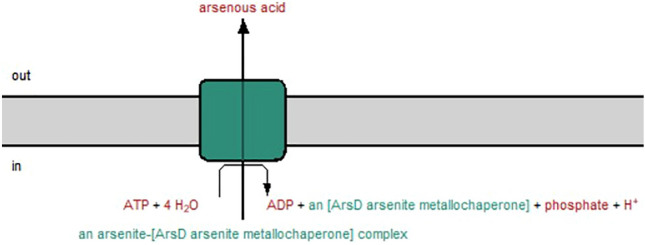
ArsD transporter pathway for the excretion of As (III) generated through the Pathway tool. The pathway is similar to the arsA pathway which hydrolyzes the ATP to ADP. ArsD is a metallochaperone complex, which involves both arsA and arsB transporter. The lack of ATP may have contributed to failure of excretion. As a result, most As (III) accumulated in the cell

The whole-genome sequence of *Nostoc* was analysed through the Pathway tool. From the results generated through the Pathway tool, *Nostoc* consists of multiple pathways related to As (III). The main pathways or compounds found in the *Nostoc* related to As (III) were (1) direct extrusion of arsenic through arsenical pump-driving ATPase (arsA) (Fig. 9), (2) extrusion of arsenic through arsA or arsB transporter which involved the arsenite metallochaperone (ArsD) complex (Fig. 10), and (3) methylation of As (III) to S-adenosyl-L-methionine (SAM) and glutathione using arsenite methyltransferase (ArsM) which formed arsenic triglutathione as a less toxic arsenic product.

### Primers expressed by *Nostoc*

Four sets of primers were designed to identify the possible genes regulated by *Nostoc* when treated with As (III) for 24 days. The primers are selected as stated in Table 3 according to the possible pathways predicted from the whole-genome sequence. Past research has shown As (III) oxidizes to As (V) and reduces arsenic toxicity. Hence, the *Acr3* gene was designed to identify the presence of As (V) in the cells.

**Table 3:** Details of arsenic-related primer sets designed

Primer set	Gene	Gene size (bp)	Function	Primer sequence	Justification behind gene selection
1	Arsenical resistance protein, Acr3	558	Arsenate mycothiol formation	F: GGTGAAGCCGTTTTCCATGG R: CGATTGAGCAGGTAGGCGAT	To identify the formation of As (V) through oxidation in the *Nostoc* cell
2	Arsenical pump-driving ATPase, ArsA	304	Arsenite extrusion pump	F: CGATGATGCAGCTCCAGGAT R: ACAAGGGCGAGCAGTTTCTC	To identify the pathway of As (III) removal
3	Arsenite methyltransferase	531	Methylation of Arsenite	F: TGTCGAACCTCGTCAACATC R:AGGCCATAGACGACCAGGTA	To identify As (III) methylation process
4	Glutathione-S-transferase, GST	230	Production of GSH	F: GCCTGTCAGCAGAAGAGTGA R: GGCCTGATAGTTCAGGACCA	To identify the synthesis of GSH

The overall result illustrated that all RNA samples for both treated and control showed a clear band for the housekeeping gene (SI. 1). Among multiple arsenic-related genes and the *GST* gene, only the arsenite methyltransferase gene (SI. 2) showed a faint band in the gel.

## Discussion

### The toxic arsenic species improves microalgae growth

The overall biomass results demonstrated that arsenic has the potential to enhance the growth of *Nostoc* under phosphate-depleted conditions. High As (III) concentrations are proven to improve the biomass of *Nostoc* during the exponential phase and increase the biomass of *Chlorella* during the initial cultivation phase. As (III) is taken up into microalgae cells through aquaglyceroporins (Arora et al. 2018), while As (V) enters the microalgae cells through a phosphate transporter system (Ferrari et al. 2013). This mechanism leads to the entering of As (V) into microalgae cells will be competitive with the presence of phosphate, whereas As (III) is not affected by the presence of phosphate. Consequently, high arsenic concentration can be incorporated into the microalgae cells, thereby improving the microalgae’s growth. However, our findings indicate that when comparing conditions with and without phosphate, As (III) enhances the *Nostoc* biomass when there is a lack of phosphate compared to the control. This suggests that phosphate will reduce the effect of As (III) on the *Nostoc* biomass. In phosphate-depleted conditions, *Nostoc* cells utilize As (III) as a nutrient source (Knauer and Hemond 2000), and a high phosphate concentration does not protect cells from arsenic toxicity (Knauer and Hemond 2000). Conversely, As (III) reduces the *Chlorella* biomass when there is a lack of phosphate. This suggests that As (III) has the minimal capability to substitute the function of phosphate in *Chlorella* and could only perform effectively with high As (III) concentration during the initial cultivation days. The toxicity of As (III) led to a gradual reduction in the microalgae biomass after 2 days of cultivation. In the presence of phosphate, *Chlorella* biomass increased at lower As (III) concentration treatment, indicating that low levels of As (III) can enhance the growth of *Chlorella* with the assistance of phosphate*.*

On the other hand, As (V) leads to a decrease in *Nostoc* biomass at the concentration of 0.01 mg L^−1^ when phosphate is supplied, in contrast to the control. Our findings suggest that As (V) is toxic to *Nostoc* in the presence of phosphate, but non-toxic when there is a lack of phosphate. The ability of As (V) to enter the *Nostoc* cell with both conditions with or without phosphate indicated that phosphate does not inhibit As (V) from entering the microalgae cells through competition. The possible reason behind this could be due to the phosphate channel being able to distinguish between As (V) and phosphate. The presence of high-affinity and low-affinity phosphate systems, Pst (Martín and Liras 2021), and 2 clusters of genes for phosphate in *Nostoc* (Miyashita et al. 2015) increase the rate of phosphate uptake. As the channels might be capable of discriminating As (V) and phosphate (Wang et al. 2015), the presence of phosphate does not appear to reduce the As (V) uptake by *Nostoc*.

Conversely, the mechanism differs in *Chlorella* cells, as As (V) only improves the biomass when there is a presence of phosphate. These results align with Miazek et al. (2015), where As (V) stimulated *Chlorella* growth under low phosphate concentration. This suggests that As (V) has a toxic effect on *Chlorella* as it reduces the biomass in the absence of phosphate. The presence of phosphate reduces the toxicity of high As (V) concentration to the *Chlorella* biomass. The reason could be due to the competition between As (V) and phosphate, where phosphate reduces the arsenic entering the *Chlorella* cells. As (V) enters microalgae cells through the phosphate channel. Even though phosphate was not externally supplied to the *Chlorella* cells, the residual phosphate within the cell could be sufficient to support and maintain their growth for the control treatment. The introduction of additional arsenic resulted in a reduction in biomass, and the absence of phosphate supply does not inhibit the As (V) from entering the cells. In addition, a slight increase in *Chlorella* growth was observed with low arsenic concentration compared to high concentration on day 4. The possible reason could be due to As (V) failing to be incorporated into the *Chlorella* cell (Ferrari et al. 2013) in the absence of phosphate. Cells with sufficient phosphate may not activate the phosphate channel, preventing As (V) from entering the *Chlorella* cells after several days of cultivation.

Mixed arsenic treatments improve the growth of *Nostoc* when phosphate is lacking, but reduce biomass when phosphate is supplied. This is similar to single arsenic species treatment, where arsenic improves the biomass of *Nostoc* under phosphate-depleted conditions. This suggests that a mixed arsenic species with different concentrations does not influence the effect on *Nostoc*. In contrast, mixed arsenic treatments reduce the biomass of *Chlorella* in both the absence and presence of phosphate. This demonstrated that mixed arsenic species have a toxic effect on *Chlorella* cells, primarily due to the lack of phosphate. This further proves that the effect of arsenic on microalgae cells is associated with the presence of phosphate. The presence of phosphate may influence the entry of arsenic into *Chlorella* cells, whereas the lack of phosphate allows arsenic to enter the cells and reduce *Chlorella* biomass.

The overall comparison between the effects of As (III) and As (V) on *Nostoc* and *Chlorella* indicates that the mechanisms involving arsenic differ between the two microalgae strains. Under phosphate-depleted conditions, arsenic enhances the biomass of *Nostoc*, and contradictorily, reduces the growth of *Chlorella*.

### Arsenic supplied concentration affects microalgae growth

Among the 3 different arsenic concentrations selected in this study, the mid-arsenic concentration which is 0.10 mg L^−1^ showed an enhancement in the biomass compared to the lower and higher arsenic concentration. This outcome is unexpected, as past research demonstrated growth results are inversely proportional to the arsenic concentrations (Wang et al. 2015; Bahar et al. 2016).

Our results indicate that a concentration of 0.10 mg L^−1^ As (III) without phosphate and 0.10 mg L^−1^ As (V) with the presence of phosphate improves the biomass. Interestingly, the lower and higher arsenic concentration does not show the same effect, suggesting that microalgae growth is irrespective of the arsenic concentration supplied, similar to Das et al. (2023) which stated that *Diacronema lutheri* growth is irrespective of the arsenic concentration as it combats the effect of arsenic stress. Several reasons may explain the performance of 0.10 mg L^−1^ concentration is better be, due to (1) 0.10 mg L^−1^ concentration is not toxic to microalgae and able to improve the biomass of microalgae. A lower concentration at 0.01 mg L^−1^ is not toxic to microalgae cells but insufficient to improve the microalgae growth. (2) 0.10 mg L^−1^ arsenic concentration causes harm to microalgae cells and is sufficient to activate the defence mechanism of the cells. As the defence mechanism of microalgae cells was activated, the toxicity of arsenic was reduced. A lower arsenic concentration might harm the cell and is insufficient to activate the defence mechanism.

### *Nostoc* accumulates higher arsenic concentration without compromising its growth and able to tolerate high arsenic concentration

Among different arsenic species and concentrations, *Nostoc* uptakes higher arsenic concentration when treated with 1.00 mg L^−1^ As (III) without phosphate. This result aligns with the arsenic removal percentage result, where *Nostoc* removes the highest arsenic percentage under this treatment. These results hypothesize that the As (III) at 1.00 mg L^−1^ enhances the biomass of *Nostoc* compared to other arsenic species, primarily due to the significant arsenic uptake and removal from the medium. Although As (III) does not appear to compete with phosphate to enter the *Nostoc* cell, the arsenic concentration detected when phosphate is supplied showed lower compared to phosphate-depleted condition. Currently, there are no existing findings on the influence of phosphate on As (III) uptake by microalgae, leaving this as an unanswered question. Nevertheless, based on our results, we hypothesise that the excretion of As (III) from the cell was reduced due to the lack of phosphate. According to the predicted pathways, the arsA and arsD pathways hydrolyze ATP to excrete arsenic from the cell. When phosphate was not supplied for a period, the phosphate deficiency condition led to failure in ATP synthesis. The ATP remaining in the cell on day 24 may be insufficient to support the excretion mechanism for both arsA and arsD. This was further proven when the *ArsA* gene was not expressed in the PCR analysis result. The Acr3 pathway predicted for the transformation of As (III) to As (V) was also not regulated, indicating that oxidation of As (III) did not occur. Hence, we predicted that instead of arsenic extruding out from the cell through the ArsA pathway or oxidizing to As (V), methylation has occurred and reduces the toxicity of As (III). As (III) methylate to SAM to reduce its toxicity. The methylation process which occurred on day 24 was proven through the regulation of the arsenite methyltransferase gene through the PCR analysis. As arsenic failed to be released from the cell due to the lack of ATP, the methylated arsenic will remain in the *Nostoc* cell, which is hypothesized as the main reason for the high arsenic concentration detected on day 24 and compared to the phosphate-supplied condition.

The highest arsenic uptake for *Chlorella* occurs under the treatment of 1.00 mg L^−1^ As (III) in the absence of phosphate on day 6. This explained the biomass result where a high As (III) concentration initially promotes *Chlorella* growth during the early cultivation phases. However, as the arsenic concentration uptake increases, it subsequently leads to a reduction in *Chlorella* growth. As (V) was not detected in the absence of phosphate after several days of cultivation, which also explains the growth pattern of *Chlorella*. Only a low concentration of As (V) was detected during the initial cultivation days and no arsenic was detected after several days of cultivation. This further describes the biomass of *Chlorella* treated with As (V) increase after several days of cultivation when lack of phosphate. Phosphate does not completely inhibit the uptake of As (V). However, when comparing conditions with and without phosphate, it appears that phosphate may enhance the uptake of arsenic at lower concentrations. This could explain the fluctuation in *Chlorella* biomass throughout the cultivation period.

Both *Nostoc* and *Chlorella* demonstrate higher arsenic concentration uptake with As (III) treatment under phosphate-depleted conditions. Phosphate may associate with the ArsA mechanism which highly reduces the excretion of arsenic from the cell, leading to higher arsenic concentration detected. It is also hypothesized that As (III) may enhance the *Nostoc* biomass but the reason behind this requires further analysis, while As (V) exhibits toxicity towards *Chlorella*.

### As (III) disrupts *Nostoc* membrane structure

The SEM results showing structural changes in the *Nostoc* vegetative cell provide evidence that arsenic can affect or damage the cell structure. Arsenic was not detected through SEM–EDX, likely due to the low arsenic concentration adsorbed and is below the instrument's detection limit. The observed damage on the surface structure supports that low arsenic concentration adsorbs to the *Nostoc* vegetative cell surface and causes disruption on the cell membrane. In microalgae, cell walls serve as the first barrier in metal cation uptake. The microalgae cell wall consists of several functional groups e.g., polysaccharides, protein, and lipids, which interact and adsorb arsenic via counterion interactions (Monteiro et al. 2012).

### Techno-economic implications of the application of *Nostoc* as an arsenic bioremediator

The effectiveness of microalgae in arsenic bioremediation relies on the bioabsorption capability and ease of harvesting. Selecting an appropriate microalgae strain is crucial to maximize efficiency in metal pollution remediation. According to Richards and Mullins (2013), microalgae exhibited greater potential in the removal of metals from wastewater compared to traditional nutrients. Microalgae strains i.e., *Nannochloropsis gaditana* and *Chlorella muelleri* removed 95% of metals after 10 days of cultivation. In addition, cyanobacteria *Nostoc muscorum* and *Chlorella vulgaris* demonstrated arsenic accumulation capabilities of up to 8300 mg As kg ^−1^ DW (Patel et al. 2021) and 3890 mg As kg ^−1^ DW (Leong and Chang 2020), respectively 5 times and 2.3 times higher than aquatic macrophytes, which accumulate 1543 mg As kg^−1^ (Mkandawire and Dudel 2005). This suggests that microalgae are a more suitable candidate for arsenic bioremediation compared to other organisms. Aligning with the 2030 Agenda for Sustainable Development adopted by all United Nations Member states, taking prompt action towards climate change through a sustainable method is urgent to support the needs of the current and future generations. Among the 17 goals listed, Goal 6 emphasizes ensuring the availability and sustainable management of water and sanitation for all, highlighting the importance of equitable access to safe and affordable drinking water. Enhancing water quality by reducing pollution is crucial for providing globally safe water for use.

In the comparison between cyanobacteria and green microalgae, cyanobacteria demonstrated a notable advantage over green microalgae. Our research findings indicate that the cyanobacteria *Nostoc* exhibits a superior ability to absorb higher concentrations of arsenic compared to *Chlorella*. This observation aligns with the arsenic accumulation capabilities reported by Patel et al. (2021) and (Leong and Chang 2020). In addition, *Nostoc* MUM004 demonstrated arsenic tolerance by sustaining its growth through the cultivation period. The average cell size of cyanobacteria, ranging from 1 to 100 µm (Allaf and Peerhossaini 2022), is averagely larger compared to green microalgae, which typically varies from 0.2 to 20 µm (Tragin and Vaulot 2018). The larger cell size of cyanobacteria provides a significant advantage, making them easier to harvest for bioremediation purposes. In contrast, the smaller size of green microalgae necessitates a higher energy-intensive process for their separation from the water. The larger cell size of cyanobacteria proves advantageous during the retrieval process for post-arsenic absorption in real-life applications.

## Conclusion

This study highlights *Nostoc* as a more suitable arsenic bioremediation candidate compared to *Chlorella*, as evidenced by the average higher biomass compared to the control treatment and arsenic uptake capabilities with a BCF value of 1463.6. *Nostoc* performed better at 1.00 mg L^−1^ As (III) compared to 0.01 mg L^−1^ and 0.10 mg L^−1^ in terms of biomass dry weight (0.6 g L^−1^) and arsenic BCF value. In high arsenic concentration conditions, *Nostoc* showed the ability to remove the highest arsenic percentage (75.24%) in the absence of phosphate, as compared to conditions with phosphate (67.68%) and *Chlorella* strain (21.76%). Both microalgae strains have different mechanisms involving the uptake and metabolism of arsenic. Notably, As (V) exhibited greater toxicity than As (III) towards microalgae strains, by reducing the average growth of both microalgae strains compared to the control treatment. It was also observed that the enhancement of growth by As (III) was related to the similarities in structures to the phosphate molecule. In addition, the absence of phosphate enhanced the ability of microalgae to accumulate arsenic under As (III) treatment, where only the arsenite methyltransferase gene was regulated. Thus, the current work revealed that the cyanobacteria *Nostoc* is the more potential arsenic bioremediation candidate, where its arsenic uptake ability is further enhanced under phosphate-depleted conditions. Preliminary in silico pathway analysis and gel electrophoresis studies reveal the reduction of arsenic through the downregulation of the arsenic excretion pathway.

## Data Availability

The data that support the findings of this study are available upon request.
